# On the Minimal Amount of EEG Data Required for Learning Distinctive Human Features for Task-Dependent Biometric Applications

**DOI:** 10.3389/fninf.2022.844667

**Published:** 2022-05-10

**Authors:** Carlos Gómez-Tapia, Bojan Bozic, Luca Longo

**Affiliations:** Artificial Intelligence and Cognitive Load Research Lab, Applied Intelligence Research Centre, School of Computer Science, Technological University Dublin, Dublin, Ireland

**Keywords:** biometrics, EEG, feature extraction, machine learning, deep learning, graph neural networks

## Abstract

Biometrics is the process of measuring and analyzing human characteristics to verify a given person's identity. Most real-world applications rely on unique human traits such as fingerprints or iris. However, among these unique human characteristics for biometrics, the use of Electroencephalogram (EEG) stands out given its high inter-subject variability. Recent advances in Deep Learning and a deeper understanding of EEG processing methods have led to the development of models that accurately discriminate unique individuals. However, it is still uncertain how much EEG data is required to train such models. This work aims at determining the minimal amount of training data required to develop a robust EEG-based biometric model (+95% and +99% testing accuracies) from a subject for a task-dependent task. This goal is achieved by performing and analyzing 11,780 combinations of training sizes, by employing various neural network-based learning techniques of increasing complexity, and feature extraction methods on the affective EEG-based DEAP dataset. Findings suggest that if Power Spectral Density or Wavelet Energy features are extracted from the artifact-free EEG signal, 1 and 3 s of data per subject is enough to achieve +95% and +99% accuracy, respectively. These findings contributes to the body of knowledge by paving a way for the application of EEG to real-world ecological biometric applications and by demonstrating methods to learn the minimal amount of data required for such applications.

## 1. Introduction

Among the different techniques used for measuring brain activity, Electroencephalography (EEG) is a method used for measuring voltage fluctuation inside the electrical field generated by a subject's brain. The sampling process involves placing surface electrodes onto a subject's scalp, capturing brain activity at different regions. The resulting EEG signals are non-stationary, meaning frequency values along time are not constant but variable, making them unpredictable. Recent advances in Deep Learning (DL) (Scarselli et al., 2008; Goodfellow et al., 2014; Vaswani et al., 2017) have allowed for new and improved applications of EEG signals. These applications include but are not limited to: EEG-based biometric systems (DelPozo-Banos et al., 2015), Brain-Computer Interfaces (BCIs) (Vaid et al., 2015), emotion recognition systems (Jenke et al., 2014), alertness analysis (Subasi, 2005) and medical diagnosis and progression assessment for neurological diseases such as Alzheimer's (Cassani et al., 2018) or epilepsy (Acharya et al., 2015). These systems are often composed of an automatic feature extraction layer that extracts representative features from the raw EEG signal, and a classifier for fitting a specific target feature. For example, Wilaiprasitporn et al. (2019) used a combination of Convolutional Neural Networks along with different type of temporal layers to learn distinctive features, and a fully connected layer for the purpose of classification. Similarly, Ullah et al. (2018) used a model composed of several 1-D convolutional layers for feature extraction and denoising to obtain representative features subsequently handled by a fully connected layer with a majority voting scheme.

In detail, this research focuses on biometrics, a process consisting of measuring and analyzing unique physical characteristics from humans to authenticate their unique identity. Among the methods employed for such a task, EEG has proven to be a robust biometrics method (Jayarathne et al., 2017). As pointed by Campisi and La Rocca (2014), EEG poses several advantages when compared to traditional exposed biometric modalities such as fingerprint or iris scanning. Namely: The advantage of being more robust against spoofing attacks (Revett, 2012), given the technological complexity of synthetically generating brain signals replicating the response for a specific individual. The advantage of being universal, meaning the sampling process is valid for anyone with no brain pathological conditions. Lastly, EEG poses the advantage of the aliveness detection problem not being present, meaning we can assume that the user is alive to produce brain signals sensor can read. This assumption does not necessarily hold in other biometric methods. There are two main applications for EEG in biometric systems: Person identification (PI), identifying an individual from a group of known subjects, and person authentication (PA), accepting or denying the identity of a particular individual. Both tasks' objective is to extract features with discriminating properties from EEG signals that a statistical model can later classify. Depending on the task subjects perform while their EEG recordings are taking place, it is possible to divide biometric systems into two groups, namely task-dependent (Wilaiprasitporn et al., 2019) and task-independent (Kong et al., 2018). Training a task-dependent system often means presenting the model data where all subjects perform a particular task. Whereas task-independent systems use data sampled from various tasks, making them more robust at handling unseen tasks at inference time. This work is centered around using EEG signals applied to task-dependent PI and is devoted to answering the following research question:

- **RQ:** What is the minimal amount of data required to train an affective EEG-based person identification model for biometric applications with and without an explicit feature extraction method?

The structure of this document is as follows: Section 2 provides a brief literature review on the different methods used for EEG-Based Person Identification. Section 3 explains the design and methodology, with reproducibility and replicability in mind. Section 4 discusses the results and findings and finally Section 5 summarizes these informing possible future directions.

## 2. Related Work

There exists evidence suggesting that different subjects produce different EEG responses (Marcel and Millán, 2007), with studies dating as back as the 1970's suggesting the uniqueness in EEG responses depends on each subject genetic information (Vogel, 1970; Anokhin et al., 1992). This unique property from EEG data makes it suitable for biometrics applications. However, building such applications has proven challenging to build and deploy given the non-stationary nature of EEG signals and the high quantity of noise generated during recording by, for example, muscle movements, eye blinking, electrode displacement. Additionally, there has been evidence showing that the EEG responses from an individual may vary depending on their emotions. For example, EEG responses may vary if the participant is emotionally attached to the watched person (Koelstra et al., 2009). EEG analysis often requires the extraction of high-level features fed into statistical models that automatically learn to find hidden patterns within the features. There are several different feature extraction techniques present in the literature. The feature extraction methods that are applied most often to this domain are Power Spectral Density (PSD) (Riera et al., 2007), Auto-Regressive (AR) model coefficients (Mohammadi et al., 2006; Zivot and Wang, 2006), Discrete Wavelet transform (DWT) (Guo et al., 2009; Murugappan et al., 2009), and Hilbert-Huang Transform (HHT) (Li et al., 2009). Among these techniques, wavelet-based feature extraction stands up given its performance at characterizing non-stationary data.

The available literature on person identification from EEG features dates as far as the 90's [see (Poulos et al., 1999a,b,c)]. Scholars used sets of features obtained from an AR model and a set of different Machine Learning (ML) classifiers obtaining accuracy scores ranging from 80 to 100% at classifying unique individuals from a pool of four total subjects. Mohammadi et al. (2006) trained a competitive neural network model with features obtained from an AR. In this case, scholars performed experiments training models for single-channel and multi-channel settings with up to three channels. They collected 24 s of EEG readings for 10 participants and used 15 s to train a model and a total of 24 s for testing. Results demonstrated that it was possible to obtain accuracies ranging from 80% up to 100% by using this technique on a larger pool of subjects. Brigham and Kumar (2010) used coefficients obtained for each channel from an AR model along with a Support Vector Machine (SVM) for PI. The scholars obtained testing classification accuracies close to 99% at identifying subjects from a pool of 120 subjects, suggesting that it is possible to obtain separable features from each individual. Shedeed (2011) used a combination of features obtained from applying the Discrete Fourier Transform and the Discrete Wavelet Transform followed by a voting scheme to retain the most discriminating features, which are input to a multi-layer perceptron classifier. However, their dataset was composed solely of three subjects, casting doubt on the generalization ability of their model. Thomas and Vinod (2018) used PSD features obtained from a single channel from the gamma (γ, 30–50 Hz) band along with simple correlation-based matching operations on a dataset composed of 109 subjects with an error rate of 0.0196. These results suggest that it is possible to apply a non-parametric operation over the raw EEG signal that maps input data onto a set of discriminative features for each subject.

Recent advances in DL have allowed the development of models with more expressing capabilities. Unlike approaches using hand-crafted features, DL models are often composed of two parts: a parametric feature extraction block and a classifier. The feature extraction block aims to learn representative features from the raw input signal automatically. The classifier uses the extracted features as input and predicts a target value such as a specific subject identity. Automatic feature extraction layers use spatial layers to reduce the dimensionality of the input data while trying to maintain informative features. This process usually consists of employing a combination of convolutional and pooling layers (Das et al., 2017, 2018). The input dimensionality of the data is usually not very large, given that there is a limited number of electrodes. The small input size leads to convolutional models with a relatively small number of layers, often ranging from 1 to 5 (Maiorana, 2020). Özdenizci et al. (2019) used a dataset composed of 128 subjects with readings obtained from different sessions and proposed an adversarial network from an invariant representation-learning perspective. Their goal was to create features that would be invariant between different recording sessions. Their approach consisted of an encoder convolutional layer to encode the raw signals and two classification layers. One of these layers is the identifier that predicts the correct person ID. The other one is the adversary layer that tries to predict the recording session ID. The assumption was that the encoder obtained features that the identifier could analyse but the adversary network could not, which led to session-invariant features. Additionally to the spatial layers, some approaches consider temporal layers to include temporal information in the features. Recurrent Neural Networks often include a combination of spatial and temporal layers (Chen et al., 2020). Das et al. (2019) proposed a model based on a combination of spatial layers (Convolutional) and temporal layers by employing Long Short Term Memory (LSTM). Results pointed to accuracies close to 100% on a pool of 109 subjects using the raw data from recordings of 64 channels. Wilaiprasitporn et al. (2019) proposed an alternative method using Gated Recurrent Units instead of the LSTM, achieving 99.9–100% test accuracy on the DEAP dataset (Koelstra et al., 2011) using all available channels. They also achieved 99.17% test accuracy, reducing the number of channels from 32 to 5, using their Frontal and Parietal (F3, F4, Fz, F7, and F8) configuration. Chen et al. (2020) demonstrated that it was possible to train models that automatically learn feature extraction techniques from the raw signal using spatial and temporal layers. These rich features are then injected into a classifier, where the final target feature is the subject ID. They achieved 96% test accuracy on a pool of 157 subjects sampled from four different experiments, demonstrating their model's ability to generalize.

The recent surge in interest in Graph Neural Network (GNN) architectures has also affected EEG-based applications. Applying GNNs to domains where elements and relationships exist has proven to be very effective. These applications include but are not limited to traffic flow prediction, point cloud classification, and text classification (Cui et al., 2019; Yao et al., 2019; Shi and Rajkumar, 2020). These systems also pose the advantage of implicitly stating the relationships between the different elements, making them potentially more useful for explainability purposes (Pope et al., 2019; Vilone and Longo, 2020, 2021a,b). The use of graphs for EEG-based applications remains underexplored. Zhong et al. (2020) used a Graph Convolutional Network model for EEG-based emotion recognition obtaining results comparable to other non-graph-based methods. Wang et al. (2020) used a graph to map brain functional connectivity for Participant Classification without making use of message-passing Graph Neural Networks. Their work consists of creating brain connectivity networks using multi-channel settings, node centrality, and global network metrics to compute features for the different nodes composing the graph. They prove that generated networks have significant inter-individual distinctiveness, making them suitable for biometric applications.

As these studies suggest, it is possible to obtain meaningful distinguishable patterns of behavior, as gathered by EEG signals, of a specific user, for biometric applications, with current approaches demonstrating error rates close to 0% (Wilaiprasitporn et al., 2019; Seha and Hatzinakos, 2021). However, these approaches rely on datasets composed of several minutes of recordings for each participant, sampling significant EEG windows or using a high number of training samples. Neglecting that sampling data for each participant is an expensive process that could burden real-world scenarios. Carrión-Ojeda et al. (2019) performed a similar study to find out the smallest EEG window size for a biometric system to perform subject identification. The researchers used a fixed amount of training samples per participant, leaving open the question of the minimum total time in seconds required for a PI system to be trained. Similarly, Seha and Hatzinakos (2021) developed a PI model with session-invariant features. Researchers emphasize using smaller windows than previous approaches while obtaining better results. However, they also used most of their available data for training the model, still not focusing on the total training dataset size but solely on the size of the epochs. These reasons motivated the focus on this work to provide hindsight on the minimal amount of affective-based EEG data required from each subject to train task-dependent biometric models. This minimal amount is a combination of the number of samples used for training and the window size for each sample. An exhaustive experiment have been designed, and described in the next section.

## 3. Design and Methodology

**Figure 1:**
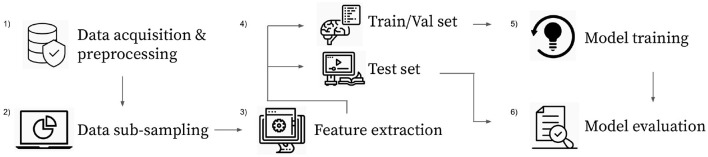
Single experiment pipeline. (1) Read raw data, remove baseline recording and pre-process. (2) Sample (60 × 128)/*w* EEG windows with size *w* for each video. (3) Extract features from all windows individually and normalize. (4) Split dataset into train, validation and testing(*tr*_*s*_/ 100/ *s* − *tr*_*s*_ − 100). (5) Train chosen model using train and validation datasets. (6) Evaluate trained model on the testing dataset. Record experiment parameters, training metrics, and testing accuracy.

This research work is devoted to understanding the minimal EEG data required to identify unique discriminative person-specific features. In order to tackle this goal, the experiment includes a combination of different training data sizes, which are defined based on the size of the sampled EEG windows and the number of training samples available per participant. In detail, these experiments include the training of models by employing several training data sizes along with different machine learning techniques, including a *Logistic Regressor* (LR), *Multi-Layer Perceptron* (MLP), *1-D Convolutional Neural Network* (CNN), and a Graph Convolutional Network (*Graphconv*) and a set of feature extraction methods, namely Power Spectral Density (PSD) and Wavelet Energy (WE). Figure 1 depicts the experimental pipeline for a given configuration. Each experimental configuration is used 10 times with different, randomly sampled data to obtain results distributions. Each pipeline aims to train and evaluate a classification model that can perform Person Identification given a sub-sampled window of EEG recordings. Training a discriminative model with data for a pool of *p* participants that automatically learns to differentiate input features depending on the participant from whom the features have been sub-sampled. More formally, the goal is to approximate a parametric function *f*_σ_ that maps an input feature matrix **X** to a vector of predicted probabilities **p** with one entry per known participant. ***p*_*i*_** represents the probability of the input features belonging to the subject with ID *i*. All together we can represent it as $f_{\sigma }\left(\text{X}\in {\mathbb{R}}^{n\times d}\right)\to {\text{p}}_{s}\in {\left[0,1\right]}^{p}$ where *n* indicates the number of EEG channels used, *d* depends on the feature extraction method and denotes the number of obtained features per channel and *p* is the total number of participants. Models are trained and validated on a randomly sub-sampled portion of the complete dataset, where there are *tr*_*s*_ training samples and 100 validation samples for each user. The evaluation process for a trained model consists of making predictions on the remaining unseen data and measuring classification accuracy. In detail, classification is correct when the highest value for a predicted probability vector ***p*_*s*_** matches the ID of the subject the recordings were sampled from.

We hypothesize that by running a set of experimental configurations and evaluating the results, it is possible to provide an answer to the research question stated in Section 1. The overall experiment, consisting of the different configuration settings, is performed to test the following hypothesis:

- **H1:** IF different affective EEG-based person identification models are trained using a variable dataset size (number of training samples and window sizes) and pre-process data extracting wavelet energy or power spectral density
  THEN it exists a minimum amount of data that can lead these models to achieve 95% and 99% of accuracy, and this amount is significantly lower than the amount associated to the same models trained without the pre-processing methods.

In the context of training person identification models, the minimum required amount of data is defined based on the total number of train EEG windows used (*tr*_*s*_) and the size for each window in seconds (*w*/128). The minimal amount of data and the best learning approach and feature extraction combination can be determined by comparing the result distributions and picking the experimental configuration able to obtain +95% and +99% accuracy on unseen data with the least amount of total training data. Moreover, performing a Mann–Whitney *U*-test over the results distributions where training data time is the same but the window size differs would answer whether it is better to have fewer oversized windows or many smaller ones.

### 3.1. Data Acquisition and Pre-processing

The experiment uses the dataset for emotion analysis using EEG, physiological, and video signals (DEAP) (Koelstra et al., 2011). It consists of a set of EEG signals and physiological and face information from 32 different participants, recorded as they watched 40 one-minute music videos meant to trigger different kinds of emotions, making this dataset task-dependent in general and affective-based in particular. The DEAP dataset poses the advantage of being sampled under naturalistic, ecological conditions. Instead of evoking a certain kind of response in a short time window, the DEAP dataset provides a continuous stream of EEG data that better resembles a real-world environment.

Thirty-two electrodes (*n* = 32) were positioned using the international 10-20 system. The current research study only considers the EEG signals. The DEAP dataset has a total of 1,280 segments (32 participants × 40 videos), where each segment consists of 32 channel readings, each with 3 s of baseline signal followed by the main recordings 60 s. The pre-processing stage consists of applying a set of pre-processing techniques to the original EEG recordings, including:

- Downsample to 128 Hz
- High-pass filter to 4.0 Hz
- Low-pass filter to 45.0 Hz.

**Figure 2:**
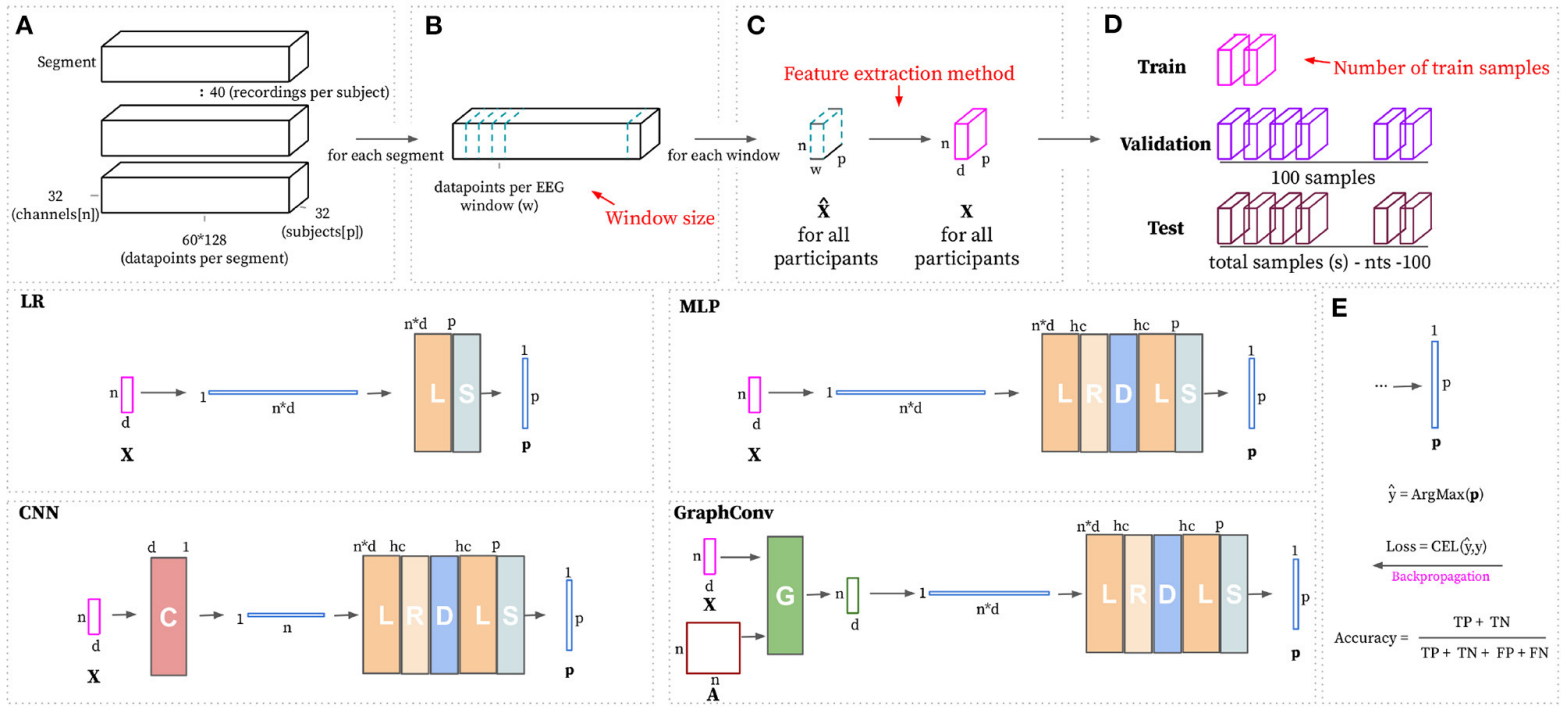
The top part of this figure shows the hyperparameters (in red) that can be modified to build the train, validation, and test datasets starting from the pre-processed segments. **(A)** Complete dataset visualized as segments. There exists one segment per video. The shape of each segment is defined based on the total number of channels (*n*), the number of subjects(*p*), and the total number of data points per video with baseline removed (60 s at 128 Hz). Panel **(B)** shows how the EEG windows are formed based on the window size (*w*). **(C)** Transforms pre-processed windows $\hat{\text{X}}$ using the feature extraction method that is set as hyperparameter. **(D)** Randomly samples *tr*_*s*_, 100 and total samples(s) - *tr*_*s*_ - 100 for train, validation and testing, respectively, per participant. The bottom part of this figure shows the considered learning approaches, with non-linear approaches having a *hc* hyperparameter for deciding the number of hidden channels. **(E)** Subject ID prediction ŷ is calculated by choosing the index of the maximum value in the predicted probability vector **p**. Training loss is computed using CELF (Equation 5). Evaluation is calculated as the number of correct predictions divided by the total number of predictions.

A band-pass filter is applied in order to extract the main frequencies of EEG human waves (Abhang et al., 2016) (Theta [4–8 Hz], Alpha [8–12 Hz], Beta [12–35 Hz], Gamma), dismissing the Delta (0.5–4 Hz) band due to it being associated with sleep activity (Amzica and Steriade, 1998). Non-parametric down-sampling is used to compress the data size. The first 3 s representing baseline recordings are removed from the rest of the signal and a sliding, non-overlapping window of varying size *w* is applied in order to split each segment into (60*128)/*w* windows (see Figure 2B). After the pre-processing steps, the total number of samples *s* is equal to 32 (participants) × 40 (videos) × (60*128)/*w* (windows per segment). Each pre-processed window $\hat{\text{X}}\in {\mathbb{R}}^{n\times w}$ consists of readings for *n* channels with *w* readings for each of them. The DEAP dataset performed its sampling with the following electrodes: [Fp1, AF3, F7, F3, FC1, FC5, T7, C3, CP1, CP5, P7, P3, Pz, PO3, O1, Oz, O2, PO4, P4, P8, CP6, CP2, C4, T8, FC6, FC2, F4, F8, AF4, Fp2, Fz, Czz].

### 3.2. Feature Extraction

All windows sub-sampled from the original segments are input to the feature extraction block. This block extracts a set of discriminating normalized features **X** from a pre-processed window $\hat{\text{X}}$ (see Figure 2C). This research work considers two feature extraction methods: Power Spectral Density (PSD) and Wavelet Energy (WE). The rationale for selecting these two techniques is that wavelet-based methods are often applied to characterize non-stationary signals, hence making it suitable for EEG data (Guo et al., 2009; Chai et al., 2017). On the other hand, using power spectral density to characterize EEG data is widely used in literature, given their high expressive power at characterizing all kinds of signals (Carrier et al., 2001). PSD features are based on the Fourier Transform (FT), whereas WE features are computed using the Wavelet Transform (WT). These transforms are invertible frequency decompositions of the original signal. The FT is often more suitable for stationary signals. In contrast, the WT better captures the characteristics of non-stationary data (Sifuzzaman et al., 2009). The main difference between FT and WT is that FT is localized within the frequency domain, and WT is both within frequency and time domains. The WT includes information about the point in time the frequencies occurs, whereas the FT does not. Whether time information makes a difference for biometric purposes is subject to study.

**Figure 3:**
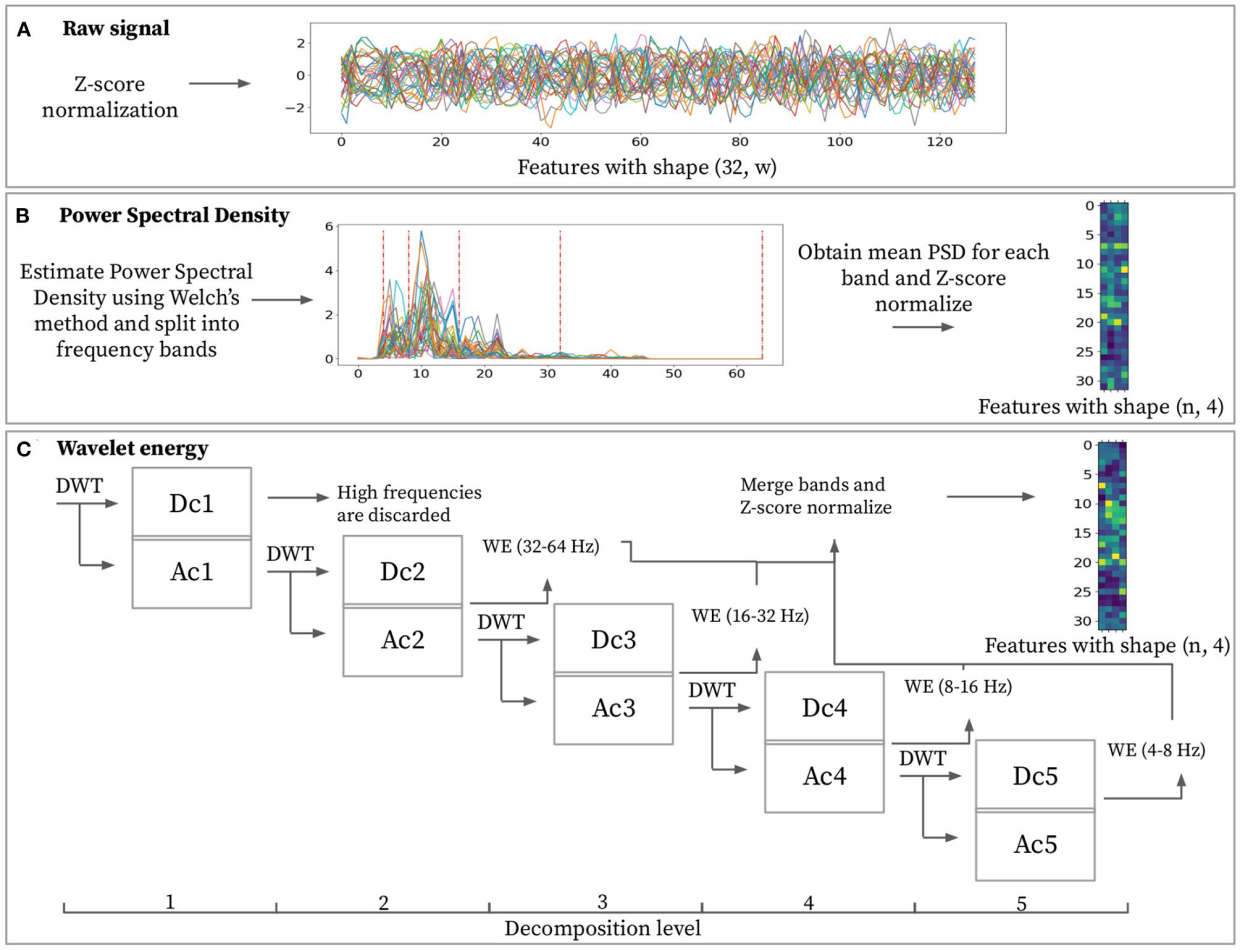
Feature extraction methods for a given sample with window size *w* = 128. **(A)** Raw signal features consist of z-score normalized power over time. **(B)** Power spectral density features are obtained by averaging power spectral density from each frequency band ([4–8, 8–16, 16–32, 32–64 Hz]) then z-score normalizing sample-wise. **(C)** Discrete wavelet transformation (DWT) is applied to the input signal and then recursively over the approximation coefficients. It is possible to extract the wavelet energy feature vectors using the wavelet energy formula (Equation 1) over detail coefficients [2–5]. The final feature matrix is built based on these wavelet energy feature vectors with z-score normalization applied to them.

To investigate their respective characterization power, raw signals are used as baseline, as depicted in Figure 3A. Raw signal features represent normalized signal power over time. Applying normalization over the input signal results in feature matrix $\text{X}\in {\mathbb{R}}^{n\times d}=\left(\hat{\text{X}}-\mu_{\hat{\text{X}}}\right)/\sigma_{\hat{\text{X}}}$ with *d* = *w*.

#### 3.2.1. Power Spectral Density (PSD) vs. Wavelet Energy (WE)

The PSD represents signal power over frequencies. Spectral density estimation is performed over the input data $\hat{\text{X}}$ in order to transform the time domain signal into the frequency domain. This domain transformation is performed using Welch's method and aims at extracting the four desired frequency bands ([4–8, 8–16, 16–32, 32–64 Hz]). Using PSD, it is possible to extract any desired frequency band. This specific band choice has the objective of matching the ones obtained by performing the wavelet decomposition for obtaining the WE feature. The final feature for every frequency band is obtained by averaging the PSD from each frequency band. Computing this feature for each channel and each frequency band results in feature matrix **X** ∈ ℝ^*n* × *d*^ with *d* = 4. Each row in the feature matrix represents a channel, and each column represents a frequency band as depicted in Figure 3B.

Wavelet energy feature is computed by splitting a pre-processed window $\hat{\text{X}}$ into different frequency ranges using a series of DWTs (see Figure 3C). The wavelet transform requires the selection of the mother wavelet and the decomposition level (N). The mother wavelet acts as a filter that is applied recursively to the input signal. In order to extract highly representative features, the mother wavelet will ideally have similar characteristics as the input signal. The “db4” mother wavelet has proven to be the most effective for EEG data (Subasi, 2007) and hence was used for all experiments. Given pre-processed signals are filtered from 4 to 45 Hz, setting the maximum decomposition level equal to 5 avoids adding noise to the features since choosing a higher decomposition level would include frequencies lower than 4 Hz. Table 1 displays the frequency range obtained from each decomposition level. Each decomposition level consists of two filters and two down samples. These filters produce an approximation coefficient containing low-frequency information and a detail coefficient containing high-frequency information. By performing the wavelet energy computations on the coefficients ranging from D2 to D5 and disregarding the D1 coefficient, we expect to reduce the total noise added onto the features, as pointed by Mohammadi et al. (2017). These coefficients for each EEG window by applying the DWT recursively over the signal's approximation coefficients at different decomposition levels. The shape of the coefficients will vary for each level due to the down sample applied at each step. With all the coefficients computed, it is possible to extract the wavelet energy feature using (1)


(1) $$\begin{array}{r} e_{j}=\overset{k_{j}}{\underset{i=1}{\sum }}{\left({\text{d}}_{j}\left(i\right)\right)}^{2} \end{array}$$

where *e*_*j*_ is the wavelet energy feature for a certain channel at decomposition level *j*. *k*_*j*_ denotes the number of wavelet coefficients for decomposition level *j* and ***d*_*j*_**(*i*) is the value of the detail coefficient at point *i* for decomposition level *j*. Calculating the wavelet energy feature for all channels results in feature matrix **X** ∈ ℝ^*n* × *d*^ with *d* = 4 where, similarly to the power spectral density features, each row represents a channel obtained from an electrode, and each column represents a particular frequency band.

**Table 1:** Wavelet signal frequencies for different decomposition levels.

**Decomposed signal**	**Frequencies (Hz)**	**Decomposition level**
D1 (noise)	64–128	1
D2	32–64	2
D3	16–32	3
D4	8–16	4
D5	4–8	5

### 3.3. Learning Approaches

Experiment settings include four different learning approaches, all of them sharing an identical goal: mapping an input sample **X** to a vector of predicted probabilities **p** indicating the probability of the input sample belonging to each unique individual. There is a linear model and three non-linear models inside the four different approaches. The *Logistic Regressor* (LR) is linear, meaning the transformation between input and output features does not include a non-linear activation function. It hence is a direct mapping between input and output. The MLP acts as a baseline for non-linear approaches. The CNN model consists of a 1-D Convolutional Layer followed by an MLP (CNN) to analyse whether merging the different frequency bands into one could reduce trainable parameters without sacrificing predictive performance. Finally, the *GraphConv* model consists of a Graph Convolutional layer followed by an MLP. This latter approach aims at modeling spatial dependencies in 3-D space among the different channels. All of the non-linear approaches have an additional hyperparameter *h* ranging from 64 up to 2048 that defines the number of hidden channels for different layers as summarized in Table 2. Figure 2 displays a simplified visualization of every considered learning approach.

**Table 2:** Configuration for each experimental phase.

**Setting name**	**Values main exp**.	**Values raw exp**.	**Description**
Feature extraction method	raw, psd, wav	raw	Feature to extract from each raw sample to build the dataset
EEG window size (*w*)	0.25, 0.5, 1, 1.5, 2	0.5	Number of seconds per sub-sampled EEG window
Number of train samples (*tr*_*s*_)	1, 2, 4, 8	16, 32, 64, 128, 256, 512	Number of train samples per participant
Model	MLP, CNN, GraphConv, LR	MLP, CNN, GraphConv, LR	Model architecture
Hidden channels	64, 128, 256, 512, 1,024, 2,048	512, 1,024	Number of hidden channels within non-linear models
Dropout rate	0.25	0.5	Probability for neurons before a Dropout layer not updating their weights after a backward pass

The *logistic regressor* consists of a single linear layer mapping the input sample **X** to a vector with a length equal to the number of participants. Input feature matrix gets flattened into a vector (with length *n* × *d*), input to the linear layer. Finally, a softmax activation function gets applied over the output of the linear layer in order to convert its output into a vector of probabilities **p** with their combined values adding up to 1. The LR is a linear model meaning the output will be a linear input transformation. High testing accuracies would indicate that the classes (unique subjects in this case) are linearly separable for the input features.

The MLP has a single hidden layer with a varying number of hidden channels *h*. The input feature matrix is flattened and fed into the hidden layer, followed by a ReLU non-linear activation function. Dropout is applied after the hidden layer, essentially deactivating at random some of the neuron weight updates to reduce overfitting and improve the final model's generalization capabilities. A softmax activation function is used after the output layer to obtain the desired probability vector **p**. As opposed to the LR, the MLP is non-linear, meaning it can apply learnable non-linear transformations to the input, which could be a benefit in some cases and is subject to study.

A 1-D Convolutional Neural Network merges all features into a single value by combining the different features before feeding this into the final classifier, an MLP with varying hidden channels *h*. The convolutional layer reduces the trainable parameter count by minimizing the shape of the input features from *d* to 1. The objective of the CNN is to map the input feature matrix **X** ∈ ℝ^*n* × *d*^ into a smaller, more informative feature vector **x** ∈ ℝ^*n*^. A ReLU function gets applied to the CNN's output and after the first layer of the MLP classifier. Similar to the MLP, dropout gets applied after the hidden layer of the MLP. A Softmax function acts as a final activation after the output layer in order to obtain the probability vector **p**.

**Figure 4:**
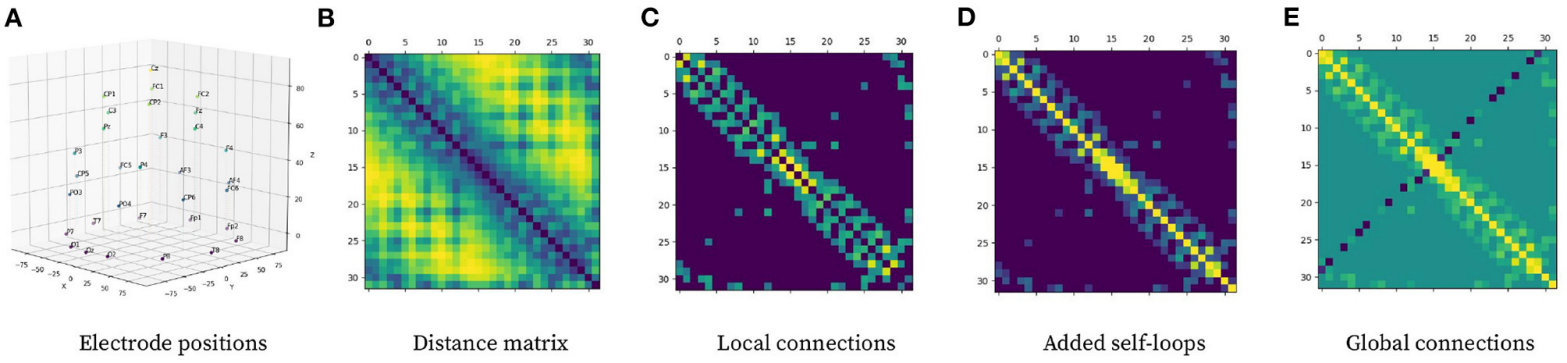
Pipeline for computing graph adjacency matrix from electrode 3-D position. **(A)** Electrode positional information in 3-D space. **(B)** Distance matrix representing Euclidean distance between electrodes. **(C)** Local connections using (2) with δ = 5. **(D)** Local connection matrix with added self-loops. **(E)** Global connections to local connection matrix with self-loops using (3).

Graph Neural Network refers to a learning approach that directly operates on graphs and can perform convolution-like operations efficiently over the input data by employing a message-passing algorithm. It is possible to model complex relationships between individual elements by modeling complex structures as graphs. A *graph* is defined as G={V,E} where V represents the set of nodes that form the graph and E refers to the set of edges connecting said nodes. {V} can be represented as a feature matrix **X** ∈ ℝ^*n* × *d*^ with *n* being the total number of nodes and *d* being the node feature dimensionality. An undirected edge set {E} can be viewed as a symmetric weighted adjacency matrix **A** ∈ ℝ^*n* × *n*^. ***A*_*ij*_** represents the importance of the relationship between node *i* and node *j*. It is possible to model a sample **X** into a graph by representing each electrode as a node, where each initial node feature $x_{i}^{t=0}\in {\mathbb{R}}^{d}$ is directly obtained from each sample feature matrix **X**. The adjacency matrix **A** is constructed based on electrode 3-D positional information (see Figure 4), and it remains constant for all samples given they follow the same 10-20 electrode placement scheme.

The first step to building the adjacency matrix is to compute local connections among electrodes. The purpose of local connectivity is to allow nodes to spread information along with their neighborhood, which should theoretically help model spatial relationships inside the graph. This process consists of constructing a distance matrix based on electrode positions and then applying the local connection formula (2); with δ = 5 denoting a calibration constant used to keep around 20% of the possible connections to maximize the efficiency of the network topology according to Zhong et al. (2020). *d*_*ij*_ represents the Euclidean distance between electrode *i* and electrode *j*.


(2) $$\begin{array}{l} {\text{A}}_{ij}=\{\begin{array}{ll} \text{min}\left(1,\frac{\delta }{d_{ij}^{2}}\right) & \text{if }min\left(1,\frac{\delta }{d_{ij}^{2}}\right)>0.1\\ 0 & \text{otherwise} \end{array} \end{array}$$

The last step is to add global connections, which aim at modeling EEG asymmetry, which are connections between distant channels in opposing parts of the brain. {*GC*} is the set of nodes that require a global connection between them ({(FP1, FP2), (AF3, AF4), (F5, F6), (FC5, FC6), (C5, C6), (CP5, CP6), (P5, P6), (PO5, PO6),(O1, O2)}) given their superior performance at modeling EEG asymmetry as stated by Zhong et al. (2020).


(3) $$\begin{array}{l} {\text{A}}_{ij}=\{\begin{array}{ll} A_{ij}-1 & \text{if }\left(i,j\right)\in \left\{GC\right\}\\ A_{ij} & \text{otherwise} \end{array} \end{array}$$

The graph layer used in this research work is similar to that presented in Morris et al. (2019), where they proposed a higher-order graph convolutional network. This architecture proposes a node update function in which nodes are updated based on their features and the features from neighboring nodes. The graph adjacency matrix defines node neighborhoods by specifying relevant connections among the different nodes that compose the graph. Node features are updated at every layer using (4), where $x_{i}^{t}$ denotes node i's feature vector at time *t*, Θ_1_ and Θ_2_ are weight matrices that are updated using loss backpropagation and N(i) indicates node i's set of neighboring nodes. The number of updates performed on every node is equal to the total number of graph convolutional layers that is included onto the architecture, either with shared or un-shared weights. Having *k* convolutional layer implies considering *k-hop* neighborhoods around each node. This experiment only considers a single graph convolutional layer due to increased performance in preliminary experiments.


(4) $$\begin{array}{l} x_{i}^{t+1}=\Theta_{1}x_{i}^{t}+\Theta_{2}\underset{j\in N\left(i\right)}{\sum }A_{ij}\cdot x_{j} \end{array}$$

The *GraphConv* architecture's tail is the same as the MLP but uses the features obtained from its graph convolutional layer as input features instead of inputting the window directly. One message-passing step gets applied to the input feature matrix **X** and this resulting feature matrix is then input to the MLP, which makes the final prediction for subject ID.

### 3.4. Training, Validation, and Testing

The training process consists of two phases: the feature extraction phase and the no-feature extraction or the raw phase. The objective of the feature extraction phase is to find out what is the minimum amount of data required to fit a Person Identification model. The raw phase is similar but aims to answer how much data is needed to train a PI model if a learned feature extraction method replaces the feature extraction step. Table 2 displays the different experimental variants . Each experiment was run 10 times with a Montecarlo sampling strategy, sub-sampling several training and validation samples from randomly selected videos at random timesteps for every participant (i.e., sample second 1 of video 23 for all participants). Combining the different experimental settings leads to 11,400 experiments for the feature extraction phase and 380 for the raw phase.

The first step is building the dataset for each desired configuration, according to the employed feature extraction method and the EEG window size. The output of such as feature extraction step is a set of feature matrices ${\text{X}}_{s}\in {\mathbb{R}}^{n\times d}$. The target feature is equal to the subject ID. Each training sample also has an associated adjacency matrix (A) that Graph-based models use and non-graph models disregard. The number of train samples per participant (*tr*_*s*_) is a hyperparameter for each experiment. Setting *tr*_*s*_ = 1 means having just one training example per participant, making this problem one-shot learning (see Figure 2D). The total number of samples per participant depends on each experimental setting, and it ranges from 1,200 (*w* = 2) to 9,600 (*w* = 0.25). The number of validation samples was kept constant at 100 for all experiments. The remaining data, unseen during training, is used for model evaluation. The number of validation samples was selected so that it is possible to keep it constant for all experiments. This was for ensuring that there are enough validation samples to avoid overfitting, and enough unseen testing samples to evaluate the models when the window size is largest (*w* = 2). Every learning approach is presented with the same training and validation data to make the model comparison fairer. Additionally, the size for each window is an experimental setting. Whether the model performs better with smaller EEG windows or bigger ones is also subject to this study. We hypothesize that having more, smaller samples would lead the model to achieve higher prediction performance with the same total amount of time per subject. Including more samples introduces the model to examples of different mental states.

All approaches were trained using the Cross Entropy Loss function (CEL, Equation 5)


(5) $$\begin{array}{l} CEL=-\frac{1}{s}\overset{s}{\underset{i=1}{\sum }}y_{i}\cdot log\left(\hat{y_{i}}\right)+\left(1-y_{i}\right)\cdot log\left(1-\hat{y_{i}}\right) \end{array}$$

Given that PI is a supervised classification problem and every architecture output are the logits of the last layer passed through a SoftMax function, along with the Adam optimizer with a fixed learning rate of 0.0005 as it empirically demonstrated superior performance in preliminary experiments. The batch size was kept constant at 32 since this is the minimum amount the training dataset could have if *tr*_*s*_ = 1. The dropout rate did not seem to make much difference in the results. It was also kept constant at 0.25 in the main experimental phase and raised to 0.5 in the raw phase to improve the model's generalization capabilities. The values for the dropout probabilities were also decided based on the preliminary experiments. An early stopping patience limit was set equal to 30 to avoid wasting resources during training. The whole training took place using 4 Tesla P100 GPUs running Pytorch and Pytorch Geometric. All experiments run in 2 weeks, each taking around 8–10 min to complete on average.

## 4. Results and Discussion

This research work's main objective is to provide insight into the minimal amount of data required for training an accurate Person Identification model. Each section provides results for an experimental phase. The first phase aims at testing the hypotheses that consider an explicit feature extraction block. This first phase also aims to test the hypothesis of having more minor windows over fewer bigger ones. The second phase tests the hypothesis with no explicit feature extraction block. Results are presented in these two first sections and interpreted in the subsequent discussion section.

### 4.1. Feature Extraction Phase Results

The first step is to analyse whether it is better to have more minor EEG windows with more available examples or bigger windows with fewer examples. Answering this question is possible by performing a Mann–Whitney *U*-test over the result distributions to compare testing accuracy for different window sizes and the number of train samples. Results show that there was a significant difference in test accuracy between experiment configurations that used (2 × 0.25 s) as opposed to (1 × 0.5 s) [*t* = 2.618, *p* < 0.01]. Similarly, experiments using (4 × 0.25 s) also performed significantly better than the ones using (1 × 1.0 s) [*t* = 3.584, *p* < 0.01]. For the 2-s combination, smaller windows again performed better, using (8 × 0.25 s) obtained better results than (1 × 2.0 s) [*t* = 4.629, *p* < 0.01]. Same was the case for (8 × 0.5 s) vs. (2 × 2.0) [*t* = 2.705, *p* < 0.01]. Supplementary Figure 1 displays the complete results for the independent *U*-Test.

**Figure 5:**
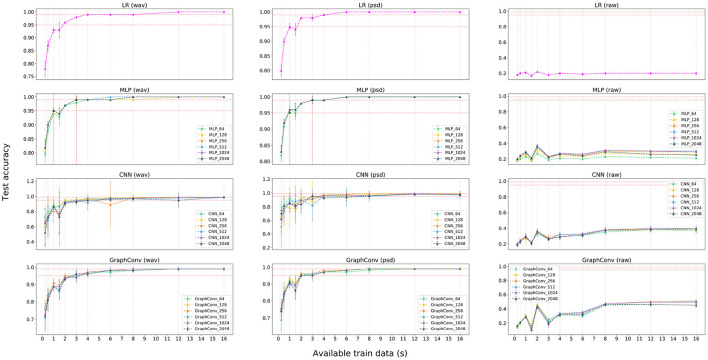
Results visualization for the feature extraction phase. Plots represent test accuracy against available training data for each participant (in seconds). Each row represents one learning approach with a legend accompanying non-linear approaches and displaying the number of hidden channels. Each column represents the employed feature extraction method, considering the raw signal as a baseline for comparison.

Supplementary Tables 1–3 display the results for the main experimental phase, listing the mean and standard deviation of the test accuracy distributions for every model. These results include a row for every learning approach and a column indicating the number of seconds of data available for each architecture during training. Figure 5 provides a visualization of these three tables for easier understanding. From these results, it is possible to observe that the LR model achieved very high performance using pre-processed features, meaning these features have a very high discriminating power that makes them linearly separable. We observe little difference in performance between the same models with different hidden channels. The MLP was able to get outstanding performance, superior to the LR but requiring more trainable parameters. The CNN achieved lower mean performance than the other models while having a significantly higher significant standard deviation. Finally, *GraphConv* had a slightly worse performance than the LR and the MLP whilst superior to the CNN. All models achieved +99% test accuracy using 8 s of data for training.

### 4.2. No Feature Extraction (Raw) Phase

**Figure 6:**
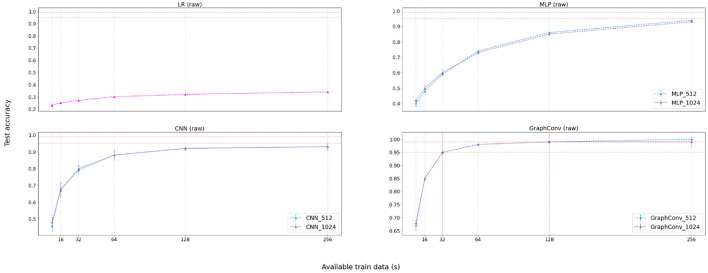
Results visualization for the no-feature extraction (raw) phase. Graphs represent accuracy against available training data for each participant (in seconds). There exists a legend accompanying non-linear approaches and displaying the number of hidden channels.

Table 2 displays the results for the raw phase. Figure 6 provides a visualization of these results for easier understanding. Results suggest that the Graph Convolutional model handles raw data better than the other models. We attribute *GraphConv's* success to its ability to extract compressed, representative features in its upper layers from the raw data alone. Similarly, the CNN model also creates features from raw data automatically. However, results demonstrate that features learned by the CNN contain less expressive information than the *GraphConv*, at least when constraining the training dataset size. Results also imply that the automatic feature extraction method is better than feeding the raw data into a classifier directly, given that both LR and MLP performed poorly when the data was not processed.

In this case, the minimal amount of data required for the best performing model to achieve 95% is 32 s per participant, with 64 EEG windows with a size of 0.5 s. As for 99% test accuracy, the model requires at least 128 s with 256 windows of size 0.5 s. In this case, it was only possible to achieve accuracy higher than 95% employing the *GraphConv* learning approach, indicating that this model's learned feature extraction step is superior to other learning approaches.

### 4.3. Discussion

Findings suggest that having more training examples per participant leads to better accuracy on test data than having fewer, bigger windows. We assume these results are because training the model with data from different trials introduces different mental states for every subject, enhancing the model's ability to recognize discriminative user features and improving predictive performance.

The minimal amount of data required to train a Person Identification model is 1 and 3 s to obtain 95% and 99% test accuracy, respectively. The model that was able to achieve 95% test accuracy and 99% with this minimal amount of data was the MLP. The results for the LR were similar to the MLP while requiring fewer parameters to be trained. Both feature extraction methods performed similarly, with PSD obtaining slightly better performance. Using raw data alone and avoiding the feature extraction step, it was possible to train a Graph convolutional model with 32 and 128 s of total data to obtain the target accuracies. These results confirm that deep learning approaches require more data than machine learning to allow robust training. We attribute the *GraphConv* model's success to its ability to learn complex feature representation automatically from raw data alone. *GraphConv* introduces spatial relationships along the different electrodes dismissed in other learning approaches. By performing a single graph convolution, the model could obtain rich features that were input to the MLP. Compared to the MLP alone, *GraphConv* shows superior performance. This superior performance indicates that the message-passing operation along the graph adds discriminative information onto the features, beneficial to the overall model predictive performance.

There exist limitations to the experiments, specifically related to the DEAP dataset. Firstly, as stated in Maiorana (2020), having single-session recordings might lead the model to learn session-specific exogenous conditions instead of personal biometric traits. However, the DEAP dataset could be considered multi-session. It consists of several independent recordings with a break at the half-mark, where electrodes are recalibrated. These facts make us believe the non-stationary nature of EEG data is preserved. Furthermore, exogenous conditions are minimized due to the experiment taking place for a long time duration. Secondly, the dataset is affective-based and sampled under naturalistic conditions. These characteristics are inherent to this dataset and should be addressed if comparing our results against any other work.

## 5. Conclusions and Future Work

Biometric systems using EEG data have seen a rise in popularity with recent advances in ML and DL, along with a deeper understanding of feature extraction methods. These techniques have allowed researchers to obtain low error rates in a wide variety of settings including using recordings sampled from different sessions and having a larger pool of unique subjects. However, there exists little work exploring how much data is needed to train accurate person identification models. This work focuses on answering this question. For this purpose a set of experiments was run comparing different train data sizes, learning approaches and feature extraction methods. The predictive performance of the trained models is then measured based on their accuracy for the unseen, test dataset. This work demonstrates that, in the context of affective EEG-based person identification, having just 1 s of data per participant is enough for training a model to achieve +95% test accuracy. Similarly, 3 s of data suffices to train a model with +99% test accuracy, if a suitable feature extraction method is chosen. The use of raw data is also explored comparing three simple models against a Graph Neural Network which is able to achieve +95% test accuracy using 32 s and +99% test accuracy using 128 s. EEG-based biometrics poses several advantages over traditional biometric systems, the main one it being secure, however, it also poses several disadvantages. Sampling EEG data is not as straightforward as scanning an iris or a fingerprint, several electrodes have to be attached to each subject and their signals must be recorded for a certain amount of time. Data collection is an expensive process. If real-world EEG-based biometric applications were to be implemented, data for every subject would have to be gathered and this data collection process should be as efficient as possible. Current research work focuses mainly on the method but fails to address this fact. These results pave the way for EEG biometrics used in real world applications, the objective being providing insight on how the data quantity changes the performance of the predictive models. Our findings suggest that in order to train an affective EEG-based biometric model, sampling data for a few seconds for every subject would be enough, as opposed to other methods found in literature which use several minutes from each subject to train their respective models. Each subject would not need to sit in a room with electrodes attached to its head for more than a few seconds. As a future direction, the number of electrodes attached to every subject to measure brain signals could also be studied, making the sampling process for this kind of application more straightforward and hence more efficient. Furthermore, task-independent methods considering a wider variety of tasks and session invariant features considering the non-stationary nature of EEG signals on multi-session datasets could also be subject to exploration.

## Data Availability Statement

Publicly available datasets were analyzed in this study. This data can be found here: https://www.eecs.qmul.ac.uk/mmv/datasets/deap/.

## Author Contributions

The main research work was developed by CG-T. LL and BB provided great insight and feedback throughout the project as well as revising the different article drafts that were developed, providing suggestions for potential improvements. All authors contributed to the article and approved the submitted version.

## Funding

This publication has emanated from research supported in part by a grant from SFI Centre for Research Training in Machine Learning at Technological University Dublin under Grant number 18/CRT/6183. For the purpose of OpenAccess, the author has applied a CC BY public copyright licence to any Author Accepted Manuscript version arising from this submission. We thank Giuliano Anselmi from IBM for granting us access to computing resources and helping us configure the IBM powerstations our models were trained on.

## Conflict of Interest

The authors declare that the research was conducted in the absence of any commercial or financial relationships that could be construed as a potential conflict of interest.

## Publisher's Note

All claims expressed in this article are solely those of the authors and do not necessarily represent those of their affiliated organizations, or those of the publisher, the editors and the reviewers. Any product that may be evaluated in this article, or claim that may be made by its manufacturer, is not guaranteed or endorsed by the publisher.
