# Supplementary material for: On the Minimal Amount of EEG Data Required for Learning Distinctive Human Features for Task-Dependent Biometric Applications
Source: Front Neuroinform. 2022 May 10;16:844667. doi: 10.3389/fninf.2022.844667 (PMC9127527; doi:10.3389/fninf.2022.844667)
Supplement: Supplementary file 1 [file Data_Sheet_1.pdf]

# *Supplementary Material*

## 1 APPENDIX

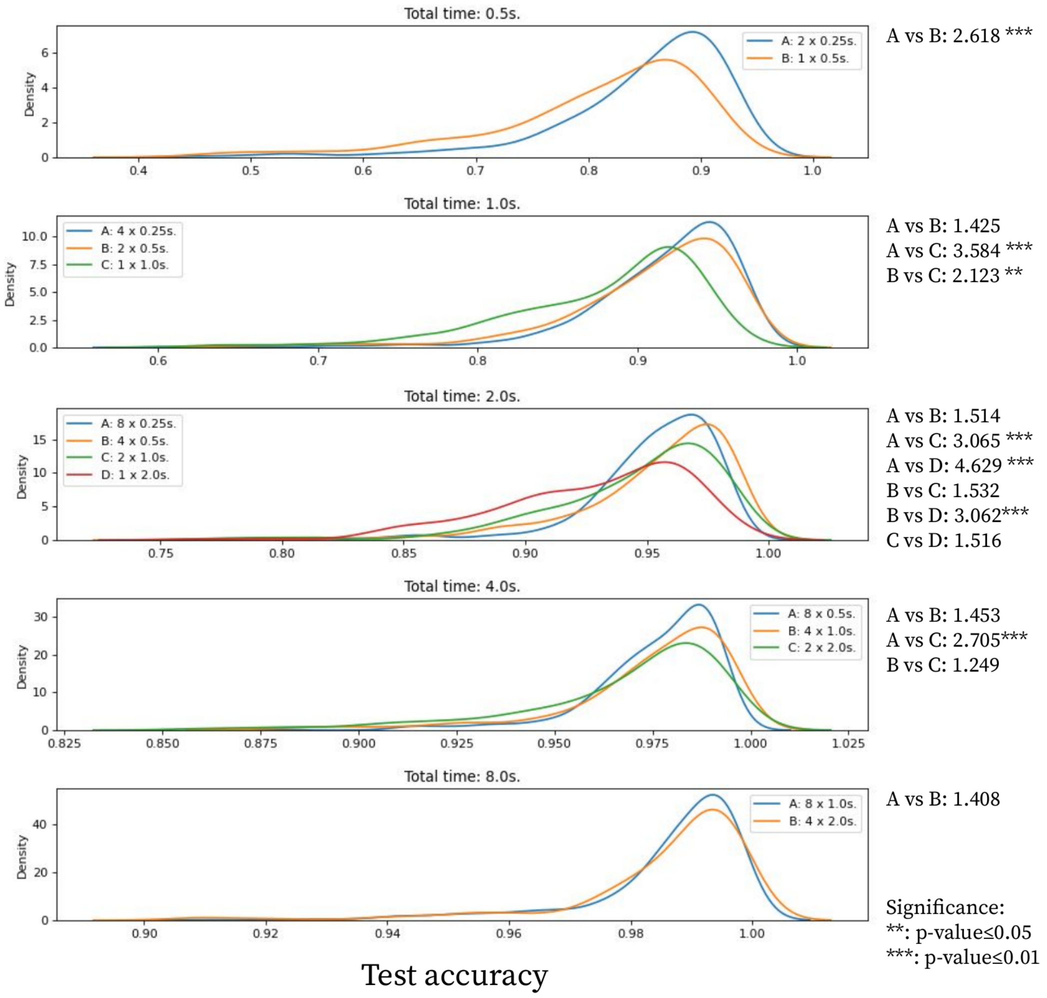


**Figure S1.** Mann–Whitney U test results for comparing results distributions for models trained with different number of EEG window sizes and number of train samples per participant. The left part right part of the figure shows

| | 0.25s. (1x0.25s) | 0.5s. (2x0.25s) | 1.0s. (4x0.25s) | 1.5s. (1x1.5s) | 2.0s. (8x0.25s) | 3.0s. (2x1.5s) | 4.0s. (8x0.25s) | 6.0s. (4x1.5s) | 8.0s. (8x1.0s) | 12.0s. (8x1.5s) | 16.0s. (8x2.0s) |
|---|---|---|---|---|---|---|---|---|---|---|---|
| **LR** | 0.78± 0.03 | 0.87± 0.02 | 0.93± 0.01 | 0.93± 0.03 | 0.96±<0.01 | 0.98±<0.01 | **0.99±<0.01** | 0.99±<0.01 | 0.99±<0.01 | **1.00±<0.01** | **1.00±<0.01** |
| **MLP 64** | 0.80± 0.03 | 0.88± 0.01 | 0.94± 0.01 | 0.93± 0.03 | **0.97±<0.01** | 0.98± 0.01 | **0.99±<0.01** | 0.99±<0.01 | 0.99±<0.01 | **1.00±<0.01** | **1.00±<0.01** |
| **MLP 128** | 0.81± 0.02 | **0.90± 0.01** | 0.94±<0.01 | **0.94± 0.03** | **0.97±<0.01** | **0.99±<0.01** | **0.99±<0.01** | 0.99±<0.01 | 0.99±<0.01 | **1.00±<0.01** | **1.00±<0.01** |
| **MLP 256** | **0.82± 0.03** | **0.90± 0.01** | **0.95± 0.01** | **0.94± 0.03** | **0.97±<0.01** | **0.99±<0.01** | **0.99±<0.01** | 0.99±<0.01 | **1.00±<0.01** | **1.00±<0.01** | **1.00±<0.01** |
| **MLP 512** | **0.82± 0.03** | **0.90± 0.01** | **0.95± 0.01** | **0.94± 0.03** | **0.97±<0.01** | **0.99±<0.01** | **0.99±<0.01** | **1.00±<0.01** | **1.00±<0.01** | **1.00±<0.01** | **1.00±<0.01** |
| **MLP 1024** | **0.82± 0.03** | **0.90± 0.01** | **0.95± 0.01** | **0.94± 0.03** | **0.97±<0.01** | **0.99±<0.01** | **0.99±<0.01** | 0.99±<0.01 | **1.00±<0.01** | **1.00±<0.01** | **1.00±<0.01** |
| **MLP 2048** | **0.82± 0.03** | **0.90± 0.01** | **0.95± 0.01** | **0.94± 0.03** | **0.97±<0.01** | **0.99±<0.01** | **0.99±<0.01** | 0.99±<0.01 | **1.00±<0.01** | **1.00±<0.01** | **1.00±<0.01** |
| **CNN 64** | 0.73± 0.09 | 0.80± 0.19 | 0.89± 0.05 | 0.73± 0.37 | 0.94± 0.02 | 0.93± 0.04 | 0.98± 0.01 | 0.97± 0.03 | 0.97± 0.04 | 0.98± 0.01 | 0.99±<0.01 |
| **CNN 128** | 0.64± 0.23 | 0.72± 0.24 | 0.87± 0.13 | 0.88± 0.04 | 0.95± 0.02 | 0.96± 0.02 | 0.96± 0.03 | 0.99± 0.01 | 0.99± 0.01 | 0.98± 0.02 | 0.99± 0.01 |
| **CNN 256** | 0.60± 0.24 | 0.73± 0.27 | 0.81± 0.15 | 0.76± 0.27 | 0.93± 0.02 | 0.95± 0.03 | 0.97± 0.02 | 0.89± 0.30 | 0.98± 0.02 | 0.99± 0.01 | 0.99±<0.01 |
| **CNN 512** | 0.67± 0.16 | 0.69± 0.19 | 0.86± 0.07 | 0.87± 0.11 | 0.93± 0.03 | 0.95± 0.02 | 0.92± 0.10 | 0.98± 0.01 | 0.97± 0.01 | 0.98± 0.01 | 0.99±<0.01 |
| **CNN 1024** | 0.65± 0.10 | 0.74± 0.14 | 0.87± 0.04 | 0.78± 0.27 | 0.92± 0.03 | 0.94± 0.04 | 0.95± 0.03 | 0.97± 0.02 | 0.98± 0.01 | 0.99± 0.01 | 0.99± 0.01 |
| **CNN 2048** | 0.52± 0.18 | 0.72± 0.16 | 0.87± 0.04 | 0.73± 0.21 | 0.91± 0.04 | 0.93± 0.03 | 0.95± 0.03 | 0.96± 0.02 | 0.97± 0.02 | 0.95± 0.09 | 0.99± 0.01 |
| **GraphConv 64** | 0.72± 0.05 | 0.81± 0.04 | 0.89± 0.01 | 0.86± 0.05 | 0.95± 0.01 | 0.94± 0.03 | 0.97± 0.02 | 0.97± 0.03 | 0.98± 0.01 | 0.99±<0.01 | 0.99± 0.01 |
| **GraphConv 128** | 0.71± 0.07 | 0.83± 0.04 | 0.89± 0.03 | 0.89± 0.03 | 0.94± 0.02 | 0.94± 0.02 | 0.97± 0.01 | 0.98± 0.01 | 0.99± 0.01 | 0.99±<0.01 | 0.99±<0.01 |
| **GraphConv 256** | 0.76± 0.05 | 0.83± 0.05 | 0.91± 0.02 | 0.89± 0.03 | 0.95± 0.01 | 0.95± 0.02 | 0.97± 0.01 | 0.98±<0.01 | 0.99± 0.01 | 0.99±<0.01 | 0.99±<0.01 |
| **GraphConv 512** | 0.71± 0.08 | 0.84± 0.03 | 0.89± 0.02 | 0.86± 0.04 | 0.94± 0.01 | 0.95± 0.02 | 0.96± 0.02 | 0.98±<0.01 | 0.99±<0.01 | 0.99± 0.01 | 0.99±<0.01 |
| **GraphConv 1024** | 0.73± 0.06 | 0.82± 0.03 | 0.89± 0.03 | 0.89± 0.04 | 0.94± 0.01 | 0.94± 0.02 | 0.97± 0.01 | 0.98± 0.01 | 0.99± 0.01 | 0.99±<0.01 | 0.99± 0.01 |
| **GraphConv 2048** | 0.72± 0.07 | 0.81± 0.06 | 0.89± 0.02 | 0.87± 0.05 | 0.93± 0.01 | 0.96± 0.02 | 0.96± 0.01 | 0.98± 0.01 | 0.98± 0.01 | 0.99±<0.01 | 0.99±<0.01 |

**Table S1.** Test accuracies for the different models tested **wavelet energy** feature extraction. Rows represent the different learning approaches, and columns represent the available training data in seconds.

| | 0.25s. (1x0.25s) | 0.5s. (2x0.25s) | 1.0s. (4x0.25s) | 1.5s. (1x1.5s) | 2.0s. (8x0.25s) | 3.0s. (2x1.5s) | 4.0s. (8x0.25s) | 6.0s. (4x1.5s) | 8.0s. (8x1.0s) | 12.0s. (8x1.5s) | 16.0s. (8x2.0s) |
|---|---|---|---|---|---|---|---|---|---|---|---|
| **LR** | 0.80± 0.02 | 0.90± 0.01 | 0.95± 0.01 | 0.94± 0.02 | **0.98±<0.01** | 0.98± 0.01 | **0.99±<0.01** | **1.00±<0.01** | **1.00±<0.01** | **1.00±<0.01** | **1.00±<0.01** |
| **MLP 64** | 0.82± 0.02 | 0.91± 0.01 | **0.96± 0.01** | 0.95± 0.02 | **0.98±<0.01** | **0.99± 0.01** | **0.99±<0.01** | **1.00±<0.01** | **1.00±<0.01** | **1.00±<0.01** | **1.00±<0.01** |
| **MLP 128** | **0.83± 0.02** | **0.92± 0.01** | **0.96± 0.01** | **0.96± 0.02** | **0.98±<0.01** | **0.99± 0.01** | **0.99±<0.01** | **1.00±<0.01** | **1.00±<0.01** | **1.00±<0.01** | **1.00±<0.01** |
| **MLP 256** | **0.83± 0.02** | **0.92± 0.01** | **0.96± 0.01** | **0.96± 0.02** | **0.98±<0.01** | **0.99± 0.01** | **0.99±<0.01** | **1.00±<0.01** | **1.00±<0.01** | **1.00±<0.01** | **1.00±<0.01** |
| **MLP 512** | **0.83± 0.02** | **0.92± 0.01** | **0.96± 0.01** | **0.96± 0.02** | **0.98±<0.01** | **0.99± 0.01** | **0.99±<0.01** | **1.00±<0.01** | **1.00±<0.01** | **1.00±<0.01** | **1.00±<0.01** |
| **MLP 1024** | **0.83± 0.02** | **0.92± 0.01** | **0.96± 0.01** | **0.96± 0.02** | **0.98±<0.01** | **0.99± 0.01** | **0.99±<0.01** | **1.00±<0.01** | **1.00±<0.01** | **1.00±<0.01** | **1.00±<0.01** |
| **MLP 2048** | **0.83± 0.02** | **0.92± 0.01** | **0.96± 0.01** | **0.96± 0.02** | **0.98±<0.01** | **0.99± 0.01** | **0.99±<0.01** | **1.00±<0.01** | **1.00±<0.01** | **1.00±<0.01** | **1.00±<0.01** |
| **CNN 64** | 0.75± 0.08 | 0.84± 0.12 | 0.93± 0.02 | 0.82± 0.16 | 0.94± 0.05 | 0.92± 0.15 | 0.97± 0.02 | 0.97± 0.05 | 0.96± 0.05 | 0.99±<0.01 | 0.99± 0.01 |
| **CNN 128** | 0.66± 0.27 | 0.81± 0.12 | 0.79± 0.29 | 0.75± 0.18 | 0.94± 0.04 | 0.87± 0.29 | 0.97± 0.02 | 0.96± 0.05 | 0.98± 0.03 | 0.99± 0.01 | 0.99± 0.02 |
| **CNN 256** | 0.51± 0.29 | 0.74± 0.21 | 0.86± 0.15 | 0.83± 0.27 | 0.88± 0.17 | 0.94± 0.07 | 0.97± 0.02 | 0.98± 0.02 | 0.99± 0.01 | 0.99± 0.01 | 0.99±<0.01 |
| **CNN 512** | 0.63± 0.24 | 0.78± 0.14 | 0.89± 0.04 | 0.88± 0.09 | 0.90± 0.13 | 0.82± 0.22 | 0.96± 0.03 | 0.94± 0.13 | 0.95± 0.07 | 0.99±<0.01 | 0.97± 0.05 |
| **CNN 1024** | 0.62± 0.19 | 0.74± 0.16 | 0.86± 0.09 | 0.79± 0.18 | 0.90± 0.13 | 0.91± 0.07 | 0.96± 0.03 | 0.97± 0.03 | 0.96± 0.04 | 0.99± 0.01 | 0.98± 0.02 |
| **CNN 2048** | 0.70± 0.10 | 0.81± 0.11 | 0.85± 0.12 | 0.82± 0.09 | 0.84± 0.13 | 0.96± 0.03 | 0.93± 0.03 | 0.94± 0.05 | 0.96± 0.02 | 0.98± 0.01 | 0.98± 0.01 |
| **GraphConv 64** | 0.76± 0.05 | 0.85± 0.03 | 0.92± 0.02 | 0.89± 0.04 | 0.96± 0.01 | 0.95± 0.03 | 0.97± 0.02 | 0.97± 0.02 | 0.98± 0.02 | 0.99±<0.01 | 0.99±<0.01 |
| **GraphConv 128** | 0.74± 0.06 | 0.86± 0.02 | 0.93± 0.02 | 0.91± 0.03 | 0.95± 0.01 | 0.96± 0.01 | 0.97± 0.02 | 0.98± 0.01 | 0.99±<0.01 | 0.99±<0.01 | 0.99± 0.01 |
| **GraphConv 256** | 0.76± 0.04 | 0.83± 0.08 | 0.92± 0.02 | 0.90± 0.05 | 0.96± 0.01 | 0.96± 0.02 | 0.98± 0.01 | 0.98±<0.01 | 0.99±<0.01 | 0.99±<0.01 | 0.99±<0.01 |
| **GraphConv 512** | 0.69± 0.07 | 0.83± 0.04 | 0.92± 0.02 | 0.89± 0.05 | 0.95± 0.01 | 0.96± 0.01 | 0.97± 0.01 | 0.98± 0.01 | 0.99±<0.01 | 0.99±<0.01 | 0.99±<0.01 |
| **GraphConv 1024** | 0.75± 0.04 | 0.85± 0.04 | 0.90± 0.03 | 0.89± 0.04 | 0.95± 0.01 | 0.95± 0.02 | 0.97± 0.01 | 0.98± 0.01 | 0.99±<0.01 | 0.99±<0.01 | 0.99±<0.01 |
| **GraphConv 2048** | 0.74± 0.05 | 0.84± 0.04 | 0.91± 0.03 | 0.86± 0.05 | 0.95± 0.01 | 0.95± 0.02 | 0.97± 0.01 | 0.98± 0.01 | 0.99±<0.01 | 0.99±<0.01 | 0.99±<0.01 |

**Table S2.** Test accuracies for the different models tested **power spectral density** feature extraction. Rows represent the different learning approaches, and columns represent the available training data in seconds.

| | 0.25s. (1x0.25s) | 0.5s. (2x0.25s) | 1.0s. (4x0.25s) | 1.5s. (1x1.5s) | 2.0s. (8x0.25s) | 3.0s. (2x1.5s) | 4.0s. (8x0.25s) | 6.0s. (4x1.5s) | 8.0s. (8x1.0s) | 12.0s. (8x1.5s) | 16.0s. (8x2.0s) |
|---|---|---|---|---|---|---|---|---|---|---|---|
| **LR** | 0.18± 0.01 | 0.20± 0.01 | 0.21± 0.01 | 0.17± 0.01 | 0.22±<0.01 | 0.18±<0.01 | 0.20± 0.01 | 0.19± 0.01 | 0.20± 0.01 | 0.20± 0.01 | 0.20± 0.01 |
| **MLP 64** | 0.18± 0.01 | 0.20± 0.01 | 0.23± 0.01 | 0.18± 0.01 | 0.27± 0.01 | 0.19± 0.01 | 0.21± 0.01 | 0.20± 0.01 | 0.23± 0.01 | 0.22± 0.01 | 0.21± 0.01 |
| **MLP 128** | 0.19± 0.01 | 0.22± 0.01 | 0.25± 0.01 | 0.19±<0.01 | 0.31± 0.01 | 0.22± 0.01 | 0.24± 0.01 | 0.23± 0.01 | 0.27± 0.01 | 0.26± 0.01 | 0.26± 0.01 |
| **MLP 256** | **0.20± 0.01** | **0.24± 0.01** | 0.27± 0.01 | 0.20±<0.01 | 0.34± 0.01 | 0.23± 0.01 | 0.26± 0.01 | 0.25± 0.01 | 0.30± 0.01 | 0.29± 0.01 | 0.29± 0.01 |
| **MLP 512** | **0.20± 0.01** | **0.24± 0.01** | 0.28± 0.01 | 0.21± 0.01 | 0.35± 0.01 | 0.23± 0.01 | 0.27± 0.01 | 0.26± 0.01 | 0.31± 0.01 | 0.30± 0.01 | 0.30± 0.01 |
| **MLP 1024** | **0.20± 0.01** | **0.24± 0.01** | 0.29± 0.01 | 0.21± 0.01 | 0.37± 0.01 | 0.23± 0.01 | 0.27± 0.01 | 0.26± 0.01 | 0.31± 0.01 | 0.30± 0.01 | 0.29± 0.01 |
| **MLP 2048** | **0.20± 0.01** | **0.24± 0.01** | 0.29± 0.01 | 0.21± 0.01 | 0.37± 0.02 | 0.22± 0.01 | 0.26± 0.01 | 0.24± 0.01 | 0.29± 0.02 | 0.26± 0.02 | 0.25± 0.01 |
| **CNN 64** | **0.20± 0.01** | **0.24± 0.01** | 0.29± 0.02 | 0.21± 0.03 | 0.35± 0.02 | 0.27± 0.02 | 0.29± 0.04 | 0.31± 0.03 | 0.35± 0.03 | 0.38± 0.02 | 0.37± 0.03 |
| **CNN 128** | 0.19± 0.02 | **0.24± 0.01** | 0.27± 0.04 | **0.22± 0.02** | 0.34± 0.02 | 0.26± 0.02 | 0.32± 0.02 | 0.32± 0.02 | 0.38± 0.02 | 0.38± 0.03 | 0.38± 0.02 |
| **CNN 256** | **0.20± 0.01** | **0.24± 0.01** | 0.28± 0.02 | 0.21± 0.02 | 0.35± 0.03 | **0.28± 0.03** | 0.32± 0.02 | 0.32± 0.01 | 0.38± 0.02 | 0.39± 0.02 | 0.40± 0.03 |
| **CNN 512** | 0.19± 0.01 | 0.23± 0.03 | 0.29± 0.02 | **0.22± 0.02** | 0.33± 0.04 | 0.26± 0.02 | 0.32± 0.03 | 0.32± 0.03 | 0.38± 0.03 | 0.40± 0.03 | 0.40± 0.03 |
| **CNN 1024** | **0.20± 0.01** | **0.24± 0.01** | **0.30± 0.03** | 0.21± 0.02 | 0.36± 0.02 | 0.27± 0.02 | 0.29± 0.04 | 0.33± 0.02 | 0.38± 0.03 | 0.40± 0.02 | 0.39± 0.04 |
| **CNN 2048** | 0.19± 0.02 | 0.23± 0.02 | 0.29± 0.03 | 0.21± 0.02 | 0.36± 0.03 | 0.26± 0.02 | 0.29± 0.03 | 0.31± 0.02 | 0.37± 0.04 | 0.38± 0.03 | 0.39± 0.02 |
| **GraphConv 64** | 0.14± 0.01 | 0.20± 0.01 | 0.29± 0.02 | 0.15± 0.01 | **0.46± 0.01** | 0.22± 0.01 | 0.31± 0.01 | 0.30± 0.02 | 0.45± 0.01 | 0.46± 0.01 | 0.45± 0.02 |
| **GraphConv 128** | 0.15± 0.01 | 0.21± 0.01 | **0.30± 0.03** | 0.16± 0.02 | **0.46± 0.01** | 0.24± 0.02 | **0.33± 0.01** | 0.34± 0.01 | **0.47± 0.02** | 0.49± 0.01 | 0.49± 0.01 |
| **GraphConv 256** | 0.16± 0.02 | 0.21± 0.01 | **0.30± 0.03** | 0.14± 0.03 | 0.45± 0.01 | 0.24± 0.03 | **0.33± 0.01** | **0.35± 0.02** | **0.47± 0.02** | **0.50± 0.01** | **0.51± 0.01** |
| **GraphConv 512** | 0.16± 0.01 | 0.21± 0.01 | 0.29± 0.02 | 0.13± 0.02 | 0.44± 0.02 | 0.24± 0.01 | **0.33± 0.01** | **0.35± 0.02** | **0.47± 0.02** | 0.49± 0.01 | 0.50± 0.01 |
| **GraphConv 1024** | 0.16± 0.01 | 0.21± 0.01 | 0.29± 0.02 | 0.10± 0.02 | 0.43± 0.01 | 0.19± 0.05 | **0.33± 0.01** | 0.34± 0.03 | **0.47± 0.02** | 0.49± 0.01 | 0.49± 0.03 |
| **GraphConv 2048** | 0.16± 0.01 | 0.21± 0.01 | 0.29± 0.02 | 0.10± 0.01 | 0.42± 0.02 | 0.20± 0.04 | 0.32± 0.03 | 0.32± 0.04 | 0.46± 0.02 | 0.46± 0.02 | 0.45± 0.03 |

**Table S3.** Test accuracies for the different models tested using **no feature extraction** method during the feature extraction phase. Rows represent the different learning approaches, and columns represent the available training data in seconds.

| | 8.0s. (16x0.5s) | 16.0s. (32x0.5s) | 32.0s. (64x0.5s) | 64.0s. (128x0.5s) | 128.0s. (256x0.5s) | 256.0s. (512x0.5s) |
|---|---|---|---|---|---|---|
| **LR** | 0.23± 0.01 | 0.25±<0.01 | 0.27±<0.01 | 0.30±<0.01 | 0.32±<0.01 | 0.34±<0.01 |
| **MLP 512** | 0.40± 0.02 | 0.48± 0.01 | 0.59± 0.01 | 0.74±<0.01 | 0.86±<0.01 | 0.94±<0.01 |
| **MLP 1024** | 0.42± 0.01 | 0.50± 0.02 | 0.60± 0.02 | 0.73± 0.01 | 0.85±<0.01 | 0.93±<0.01 |
| **CNN 512** | 0.46± 0.03 | 0.68± 0.04 | 0.79± 0.02 | 0.88± 0.03 | 0.92± 0.01 | 0.93± 0.02 |
| **CNN 1024** | 0.48± 0.03 | 0.67± 0.04 | 0.80± 0.02 | 0.88± 0.02 | 0.92± 0.01 | 0.93± 0.02 |
| **GraphConv 512** | **0.68± 0.01** | **0.85± 0.01** | **0.95±<0.01** | **0.98±<0.01** | **0.99±<0.01** | **1.00±<0.01** |
| **GraphConv 1024** | 0.67± 0.02 | **0.85± 0.01** | 0.95± 0.01 | **0.98±<0.01** | **0.99±<0.01** | 0.99± 0.02 |

**Table S4.** Test accuracies for the different models tested during the no feature extraction phase. Rows represent the different learning approaches, and columns represent the available training data in seconds.
